# Supplementary material for: Mre11-Rad50 oligomerization promotes DNA double-strand break repair
Source: Nat Commun. 2022 May 2;13:2374. doi: 10.1038/s41467-022-29841-0 (PMC9061753; doi:10.1038/s41467-022-29841-0)
Supplement: Supplementary file 1 — Supplementary Information [file 41467_2022_29841_MOESM1_ESM.pdf]

Mre11-Rad50 oligomerization promotes DNA double-strand break repair

Vera M. Kissling, Giordano Reginato[#], Eliana Bianco[#], Kristina Kasaciunaite[‡], Janny Tilma[‡], Gea Cereghetti, Natalie Schindler, Sung Sik Lee, Raphaël Guérois, Brian Luke, Ralf Seidel, Petr Cejka* and Matthias Peter*

[#] [‡] Equal contributions

* Corresponding authors

Supplementary Information

Supplementary Figures 1-7
Supplementary Tables 1-3

Supplementary Figure 1: MR head domain interactions and MR oligomerization on DNA.

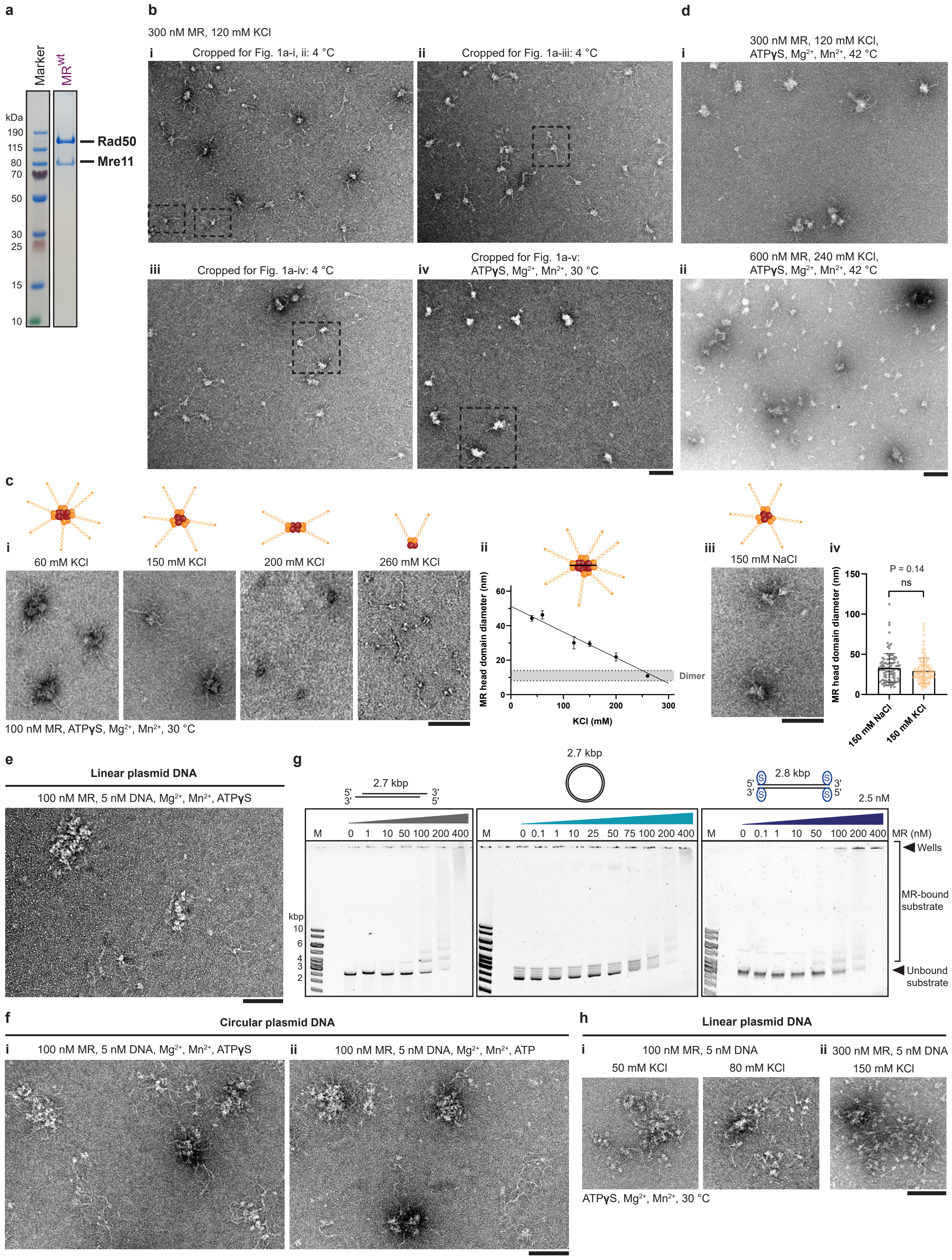

Supplementary Fig. 1. MR head domain interactions and MR oligomerization on DNA.

a Recombinant *S. cerevisiae* wild-type Mre11-Rad50 (MR^{wt}) complex used in this study. Image is representative of $n = 3$ independent purifications.

b Uncropped TEM images showing the heterogeneity of MR assemblies. $n = 3$. MR species in dashed rectangles highlighted in Fig. 1a.

c Salt-sensitivity of MR oligomers. $n = 3$. **i:** Representative collection of TEM images with cartoons indicating predominant oligomer species. **ii:** Mean head domain diameters of MR oligomers at their longest axis (MHDD; see cartoon) ± SEM as a function of KCl concentration, fitted with a linear regression. $n_{40\ mM\ KCl} = 91$ molecules, $n_{60\ mM\ KCl} = 33$, $n_{120\ mM\ KCl} = 30$, $n_{150\ mM\ KCl} = 104$, $n_{200\ mM\ KCl} = 38$ and $n_{260\ mM\ KCl} = 10$. Grey area indicates MHDD ~ 8-14 nm measured for MR dimers. **iii:** As **i** but with 150 mM NaCl. **iv:** MHDD ± SD is comparable at 150 mM NaCl and KCl (unpaired two-tailed t-test, $n_{150\ mM\ NaCl} = 96$, $n_{150\ mM\ KCl} = 104$).

d **i:** MR oligomers are larger at 42 °C than 30 °C at otherwise equal conditions (**b**-iv) or at 4 °C without co-factors (**b**-i-iii). **ii:** Higher MR concentration (600 nM) and temperature (42 °C) stabilize MR oligomers at high salt (240 mM KCl). $n = 3$.

e Uncropped TEM image of "pearls-on-a-string" zoom-inset in Fig. 1b-ii.

f **i:** Uncropped TEM image of "pearls-on-a-string" zoom-inset in Fig. 1b-iii. MR oligomerization as on linear DNA (**e**). **ii:** As **i** but with ATP. MR oligomerization on DNA is comparable with ATP and ATPγS. $n = 3$.

g EMSAs of MR with topoisomerase-relaxed circular or linearized plasmid DNA with free or streptavidin-blocked ends. Left image also shown in Fig. 1c-i. $n = 3$.

h **i:** TEM showing salt-sensitivity of MR binding to plasmid DNA at higher KCl concentrations (see Fig. 1b-ii at 30 mM KCl). **ii:** Higher MR concentration (300 nM) can stabilize MR on plasmid DNA even at 150 mM KCl. $n = 3$.

Scale bars: 100 nm (**b-f,h**). **a,g:** Uncropped gels in Source Data.

Supplementary Figure 2: MR oligomerization stabilizes MR binding on DNA.

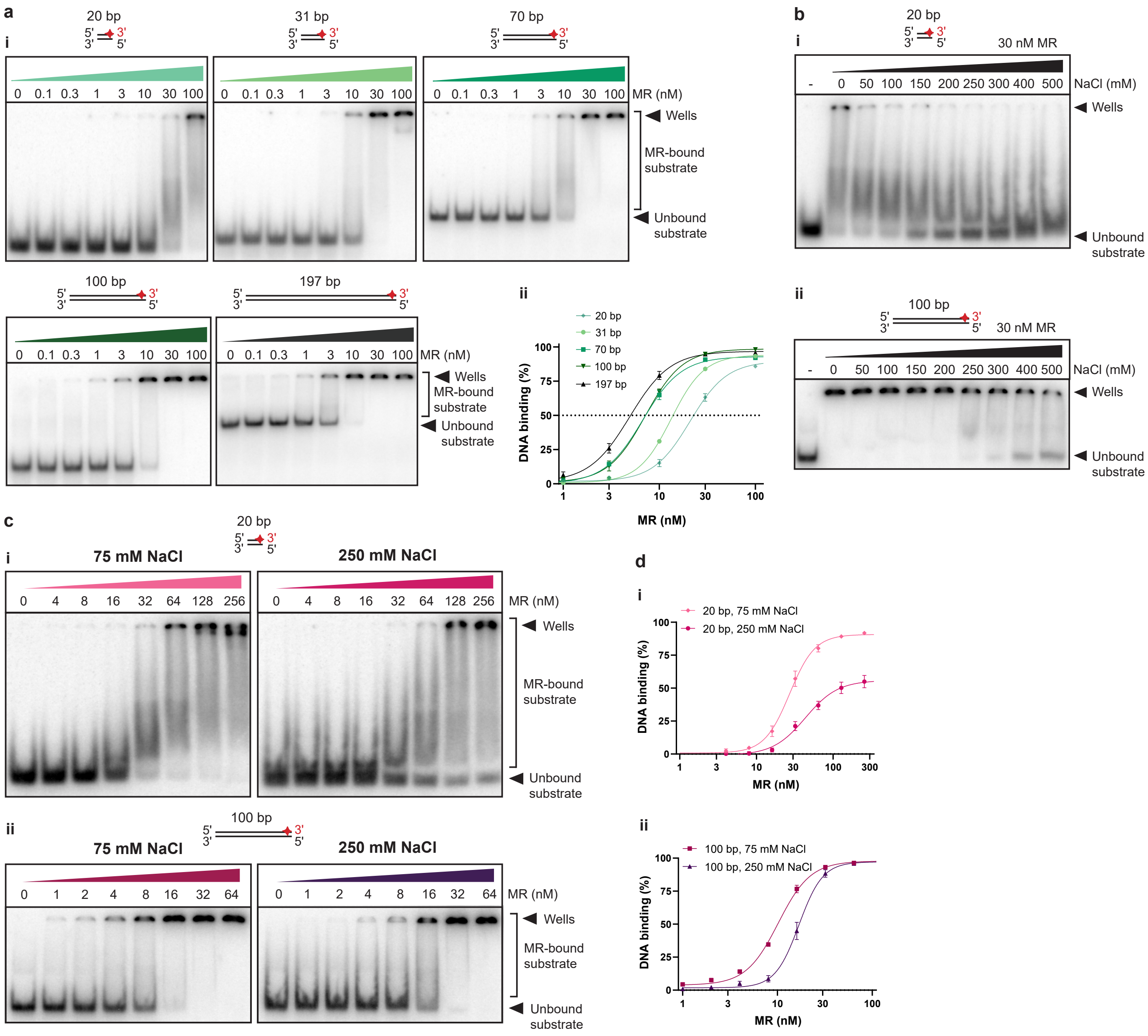

Supplementary Fig. 2. MR oligomerization stabilizes MR binding on DNA.

a EMSAs of MR binding to oligonucleotides of different lengths (100 nM base pairs) at 30 mM NaCl (**i**), and quantification (**ii**). Mean ± SEM, $n_{20,\ 31\ bp} = 4$, $n_{70,\ 100,\ 197\ bp} = 3$, sigmoidal fit. Dashed line indicates K_D as MR concentration at 50 % DNA binding; K_D per oligonucleotide plotted in Fig. 1e. Red star marks radio-label position.

b EMSAs with MR and **(i)** 20 bp or **(ii)** 100 bp-long oligonucleotides (100 nM base pairs) and various NaCl concentrations. $n = 3$. Red star marks radio-label position.

c, d EMSAs with MR and **(i)** 20 bp or **(ii)** 100 bp-long oligonucleotides (100 nM base pairs) at 75 mM or 250 mM NaCl (**c**) and quantification (**d**). Red star marks radio-label position. Mean ± SEM, $n_{20\ bp,\ 75\ mM\ KCl} = 4$, $n_{20\ bp,\ 250\ mM\ KCl} = 3$, $n_{100\ bp,\ 75\ mM\ KCl} = 5$ and $n_{100\ bp,\ 250\ mM\ KCl} = 4$, sigmoidal fit.

a-c: Uncropped gels in Source Data.

Supplementary Figure 3: MRX also oligomerizes and MRX oligomerization stabilizes MRX-DNA interaction.

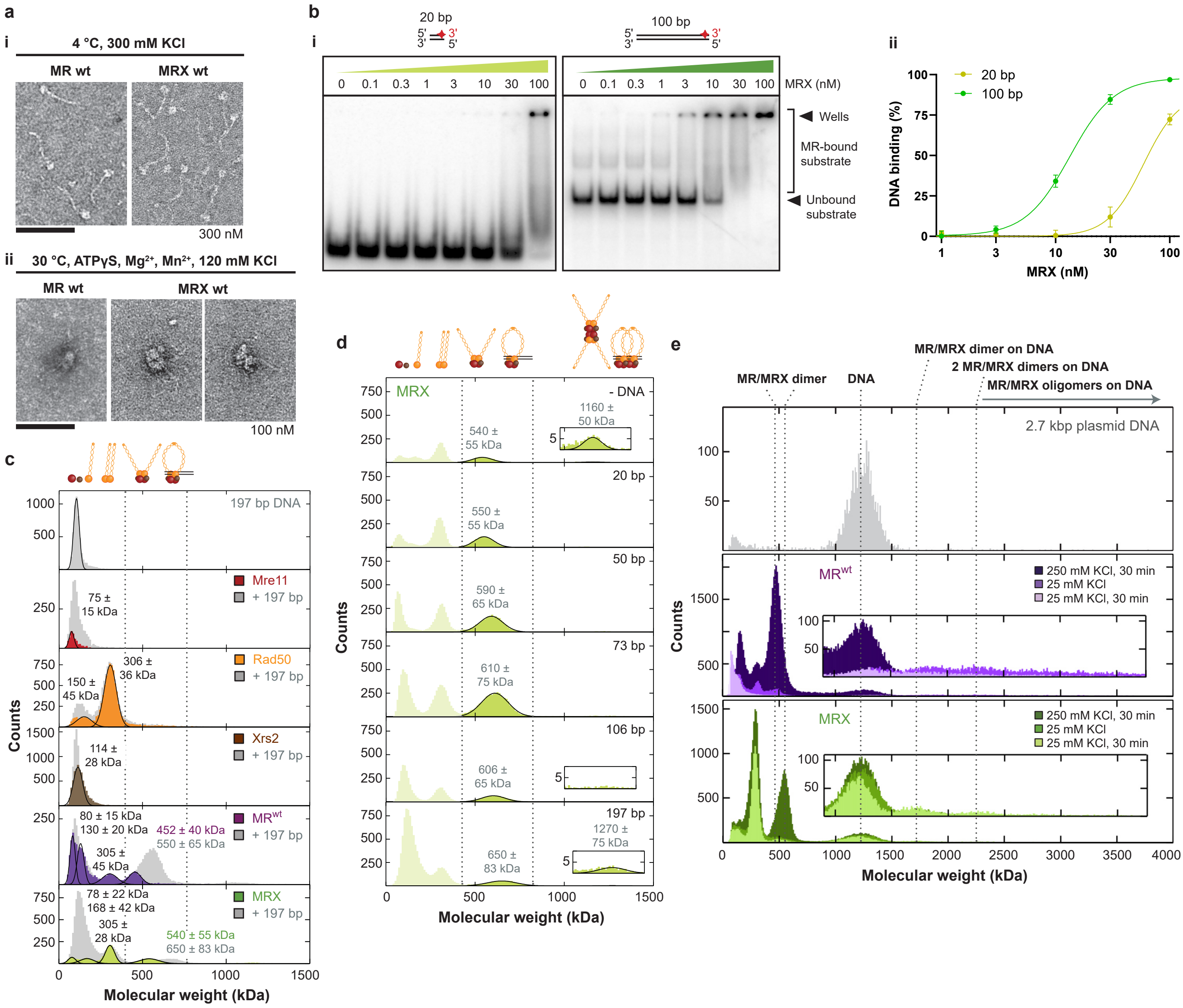

Supplementary Fig. 3. MRX also oligomerizes and MRX oligomerization stabilizes MRX-DNA interaction.

a MRX occurs as (**i**) dimers at high salt (300 mM KCl, 4 °C, no co-factors) and (**ii**) oligomerizes *via* head domain at 30 °C and 120 mM KCl with co-factors ATPγS, Mg^{2+} and Mn^{2+}, comparably to MR in TEM. $n = 3$. Scale bar: 100 nm.

b **i:** EMSAs of MRX binding to 20 bp or 100 bp-long oligonucleotides (100 nM base pairs) at 30 mM NaCl (**i**), and quantification (**ii**). MRX is stabilized on longer DNA as MR. Mean ± SEM, $n = 3$ independent experiments, sigmoidal fit. Uncropped gels in Source Data.

c Molecular mass distributions from mass photometry of Mre11, Rad50, Xrs2, MR^{wt} and MRX ± 197 bp-long DNA. Cartoons illustrate species in the peaks for MRX, exemplary also for MR^{wt}. The individual complex subunits do not bind DNA at the conditions used, but Rad50 dimerizes (measured molecular weight ± SD [MW, kDa] in black per subunit). Conversely, MR^{wt} and MRX dimers bind DNA (peaks between dashed lines; MW ± SD of MR^{wt}/MRX before [violet/green] and after DNA binding [grey]). Theoretical MW of Mre11 is 78 kDa, of Rad50 monomer 153 kDa and dimer 305 kDa, of Xrs2 96 kDa, of MR^{wt} dimer 460 kDa and of MRX dimer ($M_2R_2X_1$: 557 kDa or $M_2R_2X_2$: 653 kDa). In the MR^{wt} and MRX samples, free Mre11, and Rad50 monomer and dimer were detected (measured MW ± SD in black). $n = 3$ independent experiments. See Methods for detailed assignment of the peaks.

d Molecular mass distributions from mass photometry show better MRX binding to longer oligonucleotides (100 nM base pairs). Cartoons illustrate species in the peaks. Dashed lines frame peaks of MRX dimer ± DNA bound (MW ± SD in grey). Zoom-insets show MRX tetramers without DNA and MW range where a peak of two MRX dimers bound to one DNA molecule would be expected and was fitted where possible. Similarly to MR, larger MRX oligomers were likely formed on longer DNA beyond the detection limit of mass photometry[1], as a sudden decrease in the MRX-DNA peak areas upon binding of 106 bp or 197 bp was observed. Moreover, the MRX-DNA peak areas appear smaller than for MR (Fig. 1f), and MRX tetramers were observed in absence of DNA, suggesting that oligomerization on DNA is not only retained in the MRX complex, but even promoted. $n = 3$ independent experiments.

e Molecular mass distributions from mass photometry show the formation of MR^{wt} oligomers on plasmid DNA at 25 mM KCl (tail of events > 2500 kDa, zoom inset). After 30 min at 30 °C and low salt (25 mM KCl), as required for MR binding to oligonucleotide DNA (Fig. 1f), large nucleoprotein assemblies beyond the detection limit are formed (smaller tail of events > 2500 kDa, zoom inset). At 250 mM KCl, even after 30 min at 30 °C, MR^{wt} (~ 460 kDa) and plasmid DNA (~ 1300 kDa) are predominantly unbound. This trend was similar for MRX, but

MRX formed larger oligomers per DNA molecule than MR at low salt, thus more free DNA molecules and less of a tail of counts at high molecular weight (> 2500 kDa, zoom inset, 25 mM KCl, 30 min) could be detected. *n* = 3 independent experiments.

Supplementary Figure 4: End resection pathway reconstitutions in nuclease assays.

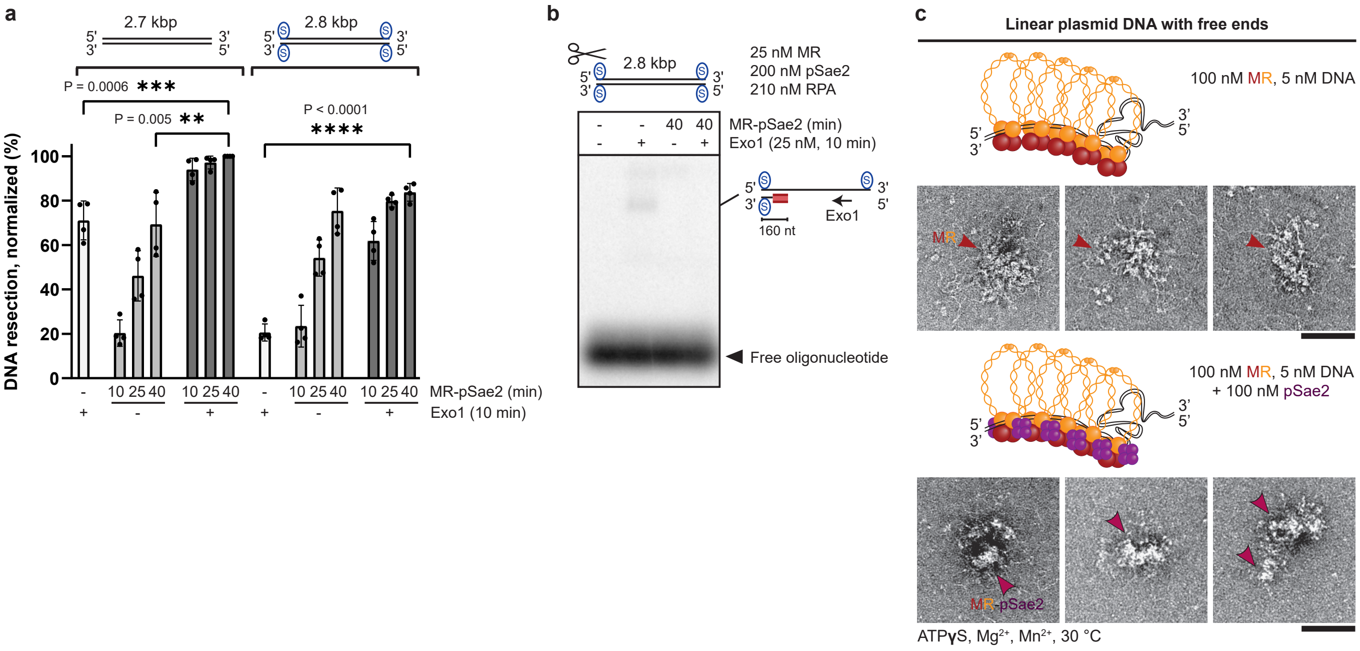

Supplementary Fig. 4. End resection pathway reconstitutions in nuclease assays.

a Quantification of nuclease assays as shown in Fig. 2a,b. Mean ± SEM, $n = 4$ independent experiments, unpaired two-tailed t-tests. Resection activity (%) was normalized such that the activity of MR-pSae2 together with Exo1 on DNA substrate with free ends at 40 min was 100 %.

b Representative nuclease assay with Exo1 on streptavidin-blocked plasmid DNA. Faint signal of resection on the complementary strand was detected with a probe (red box in cartoon) annealing 160 nt from the 3'-DNA end, suggesting that not all substrate molecules were blocked at all ends with streptavidin (see Fig. 2b). $n = 2$. Uncropped gel in Source Data.

c Representative TEM images and schematic cartoons illustrating the binding of pSae2 (violet) to MR^{wt} assemblies (red arrows and label) on plasmid DNA at 30 mM KCl. Note the formation of larger MR-pSae2 clusters on DNA (magenta arrows and label). Due to the resolution of the negative staining images, the number of molecules or their orientation drawn in the cartoons may not exactly correspond to the assembly in the micrographs. Scale bar: 100 nm.

Supplementary Figure 5: Mutagenesis of conserved Rad50 interface regulates MR oligomerization.

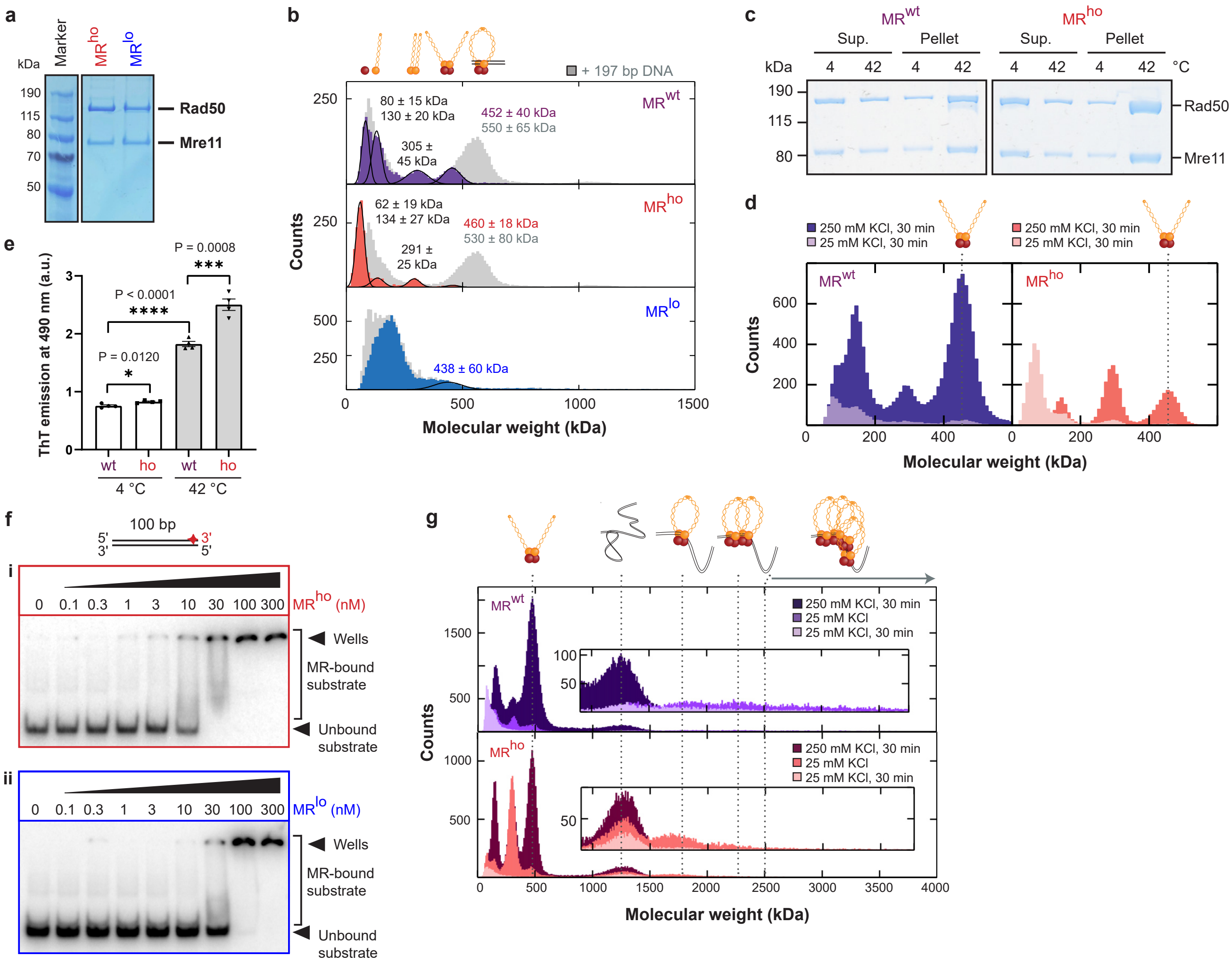

Supplementary Fig. 5. Mutagenesis of conserved Rad50 interface regulates MR oligomerization.

a Recombinant *S. cerevisiae* Mre11-Rad50$^{ho/lo}$ complexes used in this study. Image is representative of $n = 2$ independent purifications.

b Molecular mass distributions from mass photometry of MR$^{wt/ho/lo}$ ± 197 bp-long DNA. Cartoons illustrate species in the peaks. In the MR^{wt} and MR^{ho} samples, peaks of free Mre11, Rad50 monomer and Rad50 dimer could be fitted (measured molecular weight ± SD [MW, kDa] in black). Theoretical MW of Mre11 is 78 kDa, of Rad50 monomer 153 kDa and dimer 305 kDa, and MR$^{wt/ho}$ dimer 460 kDa. MR$^{wt/ho}$ dimers bound DNA (MW ± SD of MR$^{wt/ho}$ before [violet/red] and after DNA binding [grey] given), while for MR^{lo} no DNA binding was apparent (MW ± SD of unbound MR^{lo} in blue). $n = 3$ independent experiments. See Methods for detailed assignment of the peaks.

c Pelleting assays of MR$^{wt/ho}$ at 240 mM KCl (see Fig. 3f). After centrifugation, the supernatant (Sup.) and pellet of MR$^{wt/ho}$ were analyzed by SDS-PAGE. MR^{ho} forms more and/or larger assemblies than MR^{wt} that can be pelleted more efficiently, especially at higher temperature. $n = 3$.

d Molecular mass distributions from mass photometry show the formation of MR$^{wt/ho}$ oligomers without DNA at 250 mM or 25 mM KCl. MR^{ho} higher-order assemblies beyond the detection limit resist higher salt concentration, thus less MR^{ho} dimers could be detected compared to MR^{wt} (peaks with dashed line and dimer cartoon). $n = 3$ independent experiments.

e Thioflavin T fluorescence indicates larger/more beta-sheet rich, higher-order structures of MR^{ho} compared to MR^{wt} at lower and higher temperature (see Fig. 3c). Mean ± SEM, $n = 4$ independent experiments, unpaired two-tailed t-tests, a.u.: arbitrary units.

f EMSAs of (**i**) MR^{ho} and (**ii**) MR^{lo} with 100 bp-long DNA as in Supplementary Fig. 2a for MR^{wt}. Quantification is shown in Fig. 4a. $n = 3$. Red star marks radio-label position.

g Mass photometry experiments show the formation of MR$^{wt/ho}$ oligomers on plasmid DNA. Cartoons illustrate species in the peaks. The behavior of MR^{wt} is described in Supplementary Fig. 3e. MR^{ho} formed much larger nucleoprotein assemblies beyond the detection limit compared to MR^{wt} (smaller tail of MR^{ho} counts > 2500 kDa, 25 mM KCl ± 30 min). Moreover, MR^{ho} assembled larger clusters per DNA molecule than MR^{wt}, thus less unbound plasmid DNA was detected at ~ 1300 kDa with MR^{ho} (25 mM KCl, 30 min). At 250 mM KCl, MR$^{wt/ho}$ (~ 460 kDa) and plasmid DNA (~ 1300 kDa) are predominantly unbound, but MR^{ho} still oligomerizes beyond the detection limit (less dimers detected compared to MR^{wt}). $n = 3$ independent experiments. **a,c,f:** Uncropped gels in Source Data.

Supplementary Figure 6: Nuclease activities of MR mutants *in vitro*.

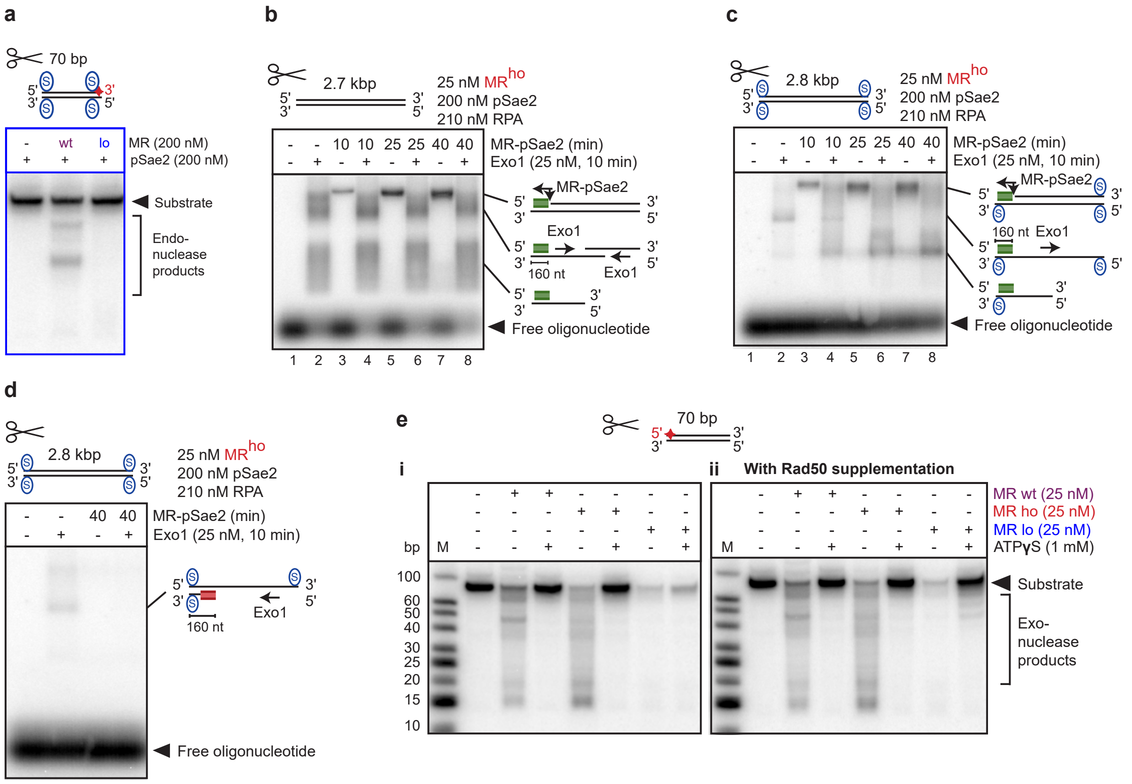

Supplementary Fig. 6. Nuclease activities of MR mutants *in vitro*.

a Representative assay demonstrating the lack of MR^{lo} endonuclease activity on streptavidin-blocked DNA with pSae2 even at higher MR^{lo} concentration (200 nM). *n* = 2. Red star marks radio-label position.

b,c Short-range end resection of MR^{ho}-pSae2 and handover to Exo1 for long-range resection (10 min, +) on plasmid DNA with free (**b**) or Streptavidin-blocked ends (**c**). Resection detection with probe (green box see cartoons) binding to the 3'-overhang produced. Image is representative of *n* = 2 experiments.

d Representative nuclease assay with Exo1 on streptavidin-blocked plasmid DNA. Faint signal of resection on the complementary strand was detected with a probe (red box in cartoon) annealing 160 nt from the 3'-DNA end, suggesting that not all substrate molecules were blocked at all ends with streptavidin. *n* = 2.

e Representative assays demonstrating exonuclease activity of $MR^{wt/ho/lo}$ on 70 bp-long DNA with co-factors (ATPγS where indicated). Bands of exonuclease products are visible for $MR^{wt/ho/lo}$. As shown in lanes 3, 5 and 7, the exonuclease of Mre11 was inhibited by ATPγS, demonstrating that all Rad50 variants bind ATP. $Rad50^{wt}$ was supplemented to account for free Mre11 subunits not in complex with Rad50 variants, which could mediate exonuclease activity insensitive to inhibition by ATP(γS) (**ii**). *n* = 2. Red star marks radio-label position.

a-e: Uncropped gels in Source Data.

Supplementary Fig. 7: MRX oligomerization modulates foci formation, DNA damage checkpoint activation and telomere maintenance.

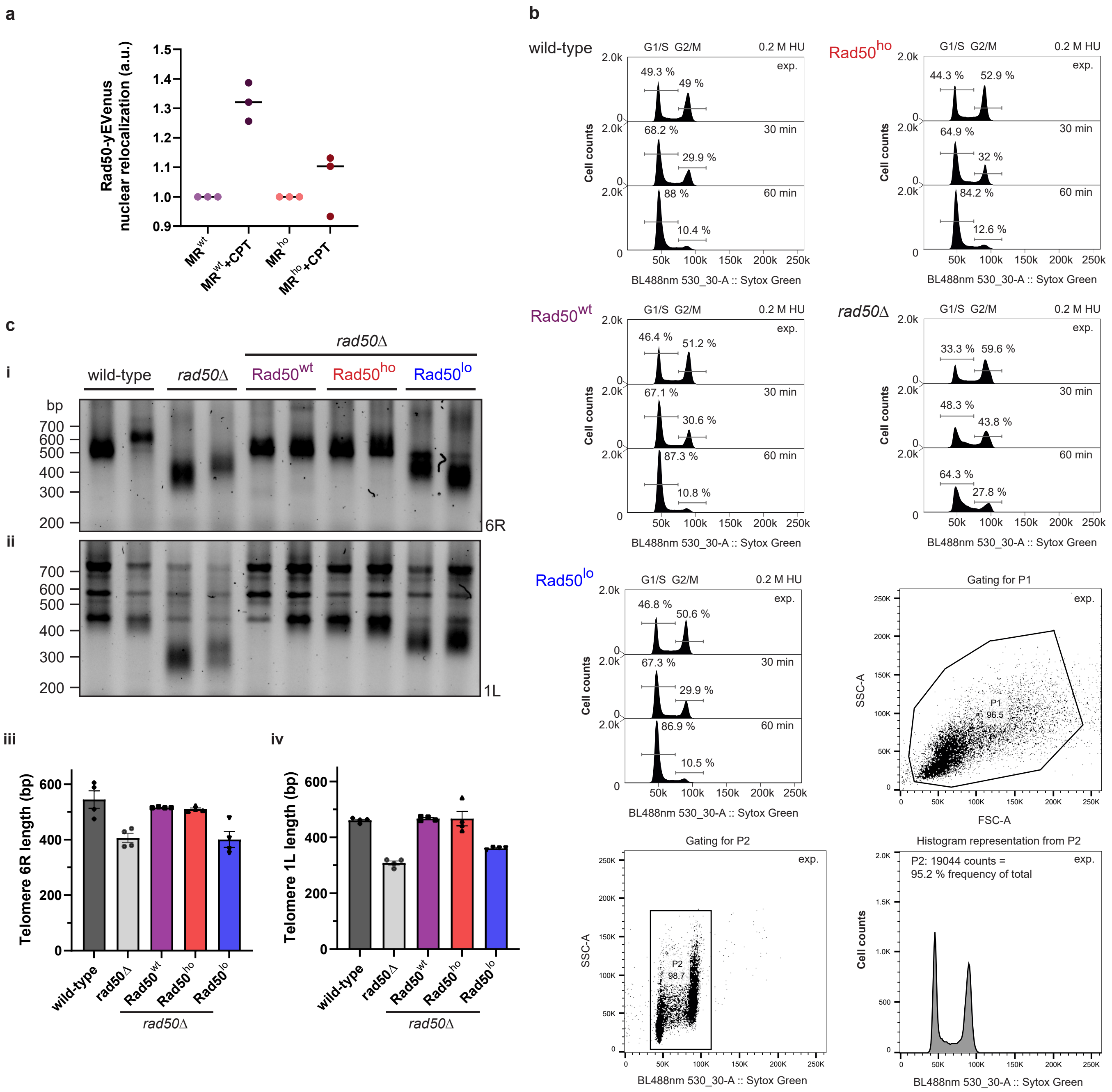

Supplementary Fig. 7. MRX oligomerization modulates foci formation, DNA damage checkpoint activation and telomere maintenance.

a Nuclear relocalization of Rad50$^{wt/ho/lo}$-yEVenus in untreated conditions and upon DSB-induction with 2 h of 200 µM camptothecin (CPT) calculated as normalized standard deviation of nuclear Rad50-yEVenus intensity from images as shown in Fig. 5e. Values were normalized to untreated conditions = 1. Median of n = 3 independent experiments is shown. Rad50^{wt} relocalizes more upon DNA damage than Rad50^{ho}, as the latter formed foci already before DSB induction and did not accumulate many more foci upon DNA damage. a.u.: arbitrary units.

b Flow cytometry-based cell cycle analysis of exponentially growing wild-type, *rad50Δ* and untagged Rad50$^{wt/ho/lo}$-expressing yeast cells in untreated (exp.) and treated conditions (0.2 M hydroxyurea, HU, 30 min and 60 min). Representative gating shown for wild-type (exp.). n = 2.

c Representative images of telomeric PCR analysis for the length of telomere 6R (**i**) and 1L (**ii**) in two clones per genotype and quantifications (**iii,iv**). PCRs were performed with oligonucleotides oBL360, oBL358 and oBL359. Fragment length was determined by using the 100 bp DNA ladder as size reference (see also Fig. 6b,c). n = 4 for each genotype; mean ± SD. Rad50^{lo}-expressing and *rad50Δ* cells have shorter telomeres than Rad50$^{wt/ho}$-expressing cells. Uncropped gels in Source Data.

Supplementary Table 1: Oligonucleotides used in this study

Name	Sequence (5' to 3')	Notes
PC216_20 nt	CGTACTCCACCTCATGCATC	Labeled oligonucleotide for 20 bp substrate
PC216_C_20 nt	GA**T**GCATGAGGTGGAGTACG	Unlabeled oligo-nucleotide for 20 bp substrate; bold **T** indicates biotin position
X12-3SC	TTGCTAGGACATGCTGTCTAGAGACTATCGC	Labeled oligonucleotide for 31 bp substrate
X12-4SC	GCGATAGTCTCTAGACAGCATGTCCTAGCAA	Unlabeled oligo-nucleotide for 31 bp substrate
PC210	G**T**AAGTGCCGCGGTGCGGGTGCCAGGGCGTGCC<br>CTTGGGCTCCCCGGGCGCGTACTCCACCTCATGC<br>A**T**C	Labeled oligonucleotide for 70 bp substrate; bold **T** indicate biotin positions
PC211	GA**T**GCATGAGGTGGAGTACGCGCCCGGGGAGCC<br>CAAGGGCACGCCCTGGCACCCGCACCGCGGCAC<br>T**T**AC	Unlabeled oligo-nucleotide for 70 bp substrate; bold **T** indicate biotin positions
BIO100C	GATGCAGGAGGCTGCTACGACCATGGCAGAAGA<br>TTATGAGGTGGAGTACGCGCCCGGGGAGCCCAA<br>GGGCACGCCCTGGCACCCGCACCGCGGCACTTA<br>C	Labeled oligonucleotide for 100 bp substrate
BIO100	G**T**AAGTGCCGCGGTGCGGGTGCCAGGGCGTGCC<br>CTTGGGCTCCCCGGGCGCGTACTCCACCTCATAA<br>TCTTCTGCCATGGTCGTAGCAGCCTCCTGCATC	Unlabeled oligo-nucleotide for 100 bp substrate; bold **T** indicates biotin position
197mer_top	**G**CTTCGTATGTTTCTCCTGCTTATCACCTTCTTGA<br>AGGCTTCCCATTCATTCAGGAACCGCCTTCTGGT<br>GATTTGCAAGAACGCGTACTTATTCGCCACCATG<br>ATTATGACCAGTGTTTCCAGTCCGTTCAGTTGTT<br>GCAGTGGAATAGTCAGGTTAAATTTAATGTGACC<br>GTTTATCGCAATCTGCCGACCACTCG	Labeled oligonucleotide for 197 bp substrate; bold **G** indicates biotin position; used for mass photometry
197mer_bottom	GCGAGTGGTCGGCAGATTGCGATAAACGGTCAC<br>ATTAAATTTAACCTGACTATTCCACTGCAACAAC<br>TGAACGGACTGGAAACACTGGTCATAATCATGG<br>TGGCGAATAAGTACGCGTTCTTGCAAATCACCAG<br>AAGGCGGTTCCTGAATGAATGGGAAGCCTTCAA<br>GAAGGTGATAAGCAGGAGAAACATACGAAG	Unlabeled oligo-nucleotide for 197 bp substrate; used for mass photometry
5'_OligoA	GGGACTGGGAAAACCCTGGCGT	Probe to detect re-section of the 5' end; the underlined sequence is complementary to plasmid DNA
3'_OligoA	GGACGCCAGGGTTTTCCCAGTC	Probe to detect re-section of the 3' end; the underlined sequence is complementary to plasmid DNA

Name	Sequence (5' to 3')	Notes
20 bp top	GACCGAGATAGGGTTGAGTG	Used for mass photometry
20 bp bottom	CACTCAACCCTATCTCGGTC	
50 bp top	GGACCACGCATAATATACCTATATCAATGGCCTC CCACGCATAAGCAGTG	
50 bp bottom	CACTGCTTATGCGTGGGAGGCCATTGATATAGGT ATATTATGCGTGGTCC	
73 bp top	GACCACCCTTTTTGATATAATATACCTATATCAAT GGGCTCCCACGCAATTCGGCAGATACGTTCTGAG GGAA	
73 bp bottom	GCCCATTGATATAGGTATATTATATCAAAAAGGG TGGTCTTCCCTCAGAACGTATCTGCCGAATTGCG TGGGA	
106 bp top	TTAGCTCCTACGCGGAACGAGACCTACCAATATA CCTATATCAATGGCCTCCCACGCATAAGCCTCGG GAGCTCGACCATCTGGTCTCATCGGACACTTAGG ACCA	
106 bp bottom	TGGTCCTAAGTGTCCGATGAGACCAGATGGTCG AGCTCCCGAGGCTTATGCGTGGGAGGCCATTGAT ATAGGTATATTGGTAGGTCTCGTTCCGCGTAGGA GCTAA	
oJT11	ATCACAAATTGAGTGGGTCGATATTAACAGAGT CACTTATGGTGACGGTGCTGGTTTA	To amplify yEVenus from pKT090
oJT12	CAATCAAAGTCTATCCCTTCGTAGATATTATGGG GTCTTTTCGATGAATTCGAGCTCG	
oJT23	ATTAGGTACCTAGTCTTGTTGCCAATAAGCACAT TTCG	To amplify --promoter-Rad50-terminator-- with restriction enzyme overhangs for cloning in pSIV URA. Used with EB181
oJT26	ATCAACTAGTATGAGCGCTATCTATAAATTATCT ATTCAGGGC	To amplify Rad50 from start until stop with SpeI/XhoI restriction overhangs for cloning into pRS416-pGAL1
oJT27	ATTACTCGAGTCAATAAGTGACTCTGTTAATATC GACCCACT	
EB181	ATCAACTAGTCTACAATACAAAAAAATAGACAA TAGTATATTTTTCTTTTGCTTGAAATT	To amplify --end promoter-Rad50-Venus-ADH1 terminator-- with restriction enzyme overhangs for cloning into pSIV URA
EB182	ATTAGGTACCCCGGTAGAGGTGTGGTCAATAAG AG	
oBL358 1L	GCGGTACCAGGGTTAGATTAGGGCTG	For Telo-PCR
oBL359	G_{18}-CGGGATCCG_{18}	
oBL360 6R	AAATGAGGACTGGGTCATGG	
oBL361 6Y’	TTAGGGCTATGTAGAAGTGCTG	

Supplementary Table 2: Plasmids used for yeast strain construction in this study

Plasmid name	Plasmid number	Description	Source
	pKT090	pFA6-link-yEVenus-Sp.His5	Euroscarf
	pNPe35	pRS416 pGal1	Mumberg, D. *et al.*[2]
	pSIV URA	pSIV URA empty vector	Wosika, V. *et al.*[3]
	pGT LEU2 mCherry	pGT LEU2 mCherry	Wosika, V. *et al.*[3]
Rad50^{wt}	pJT12	pSIVu-SpeI-prom-*rad50-yEVenus*-tADH1-KpnI	this paper
Rad50^{ho}	pJT13	pSIVu-SpeI-prom-*rad50*$^{N121A/D124N}$-*yEVenus*-tADH1-KpnI	this paper
Rad50^{lo}	pJT14	pSIVu-SpeI-prom-*rad50*$^{L116A/I119A/T127A/L128A}$ -*yEVenus*-tADH1-KpnI	this paper
	pJT22	pSIVu-SpeI-prom-*rad50*$^{N121A/D124N}$-term-KpnI	this paper
	pJT23	pSIVu-SpeI-prom-*rad50*$^{L116A/I119A/T127A/L128A}$-term-KpnI	this paper
	pJT25	pSIVu-SpeI-prom-*rad50*-term-KpnI	this paper
Rad50^{wt} OE	pJT27	pRS416 pGAL1 SpeI-*rad50*-XhoI tCYC1	this paper
Rad50^{ho} OE	pJT28	pRS416 pGAL1 SpeI-*rad50*$^{N121A/D124N}$-XhoI CYC1	this paper
Rad50^{lo} OE	pJT29	pRS416 pGAL1 SpeI-*rad50*$^{L116A/I119A/T127A/L128A}$-XhoI tCYC1	this paper

Supplementary Table 3: Yeast strains used in this study

Strain name	Strain number	Genotype	Source
Wild-type	BY4741	*Mat***a** *his3Δ1 leu2Δ0 met15Δ0 ura3Δ0*	OpenBiosystems
*rad50*Δ	yJT305	BY4741, *rad50::kanMX*	dissected from diploid collection
Rad50^{ho}-yEVenus	yJT308	BY4741, *rad50::kanMX, rad50*$^{N121A/D124N}$ *-yEVenus::URA3*	this paper
Rad50^{lo}-yEVenus	yJT311	BY4741, *rad50::kanMX, rad50*$^{L116A/I119A/T127A/L128A}$ *-yEVenus::URA3*	this paper
Rad50^{wt}-yEVenus	yJT322	BY4741, *rad50::kanMX, rad50-yEVenus::URA3*	this paper
Histone-tagged Rad50^{wt}-yEVenus	yEB371	BY4741, *rad50::kanMX*, *rad50-yEVenus::URA3, hta2::Hta2-mCherry::LEU2*	this paper
Histone-tagged Rad50^{ho}-yEVenus	yEB373	BY4741, *rad50::kanMX*, *rad50* $^{N121A/D124N}$ *-yEVenus::URA3, hta2::Hta2-mCherry::LEU2*	this paper
Histone-tagged Rad50^{lo}-yEVenus	yEB415a	BY4741, *rad50::kanMX*, *rad50* $^{L116A/I119A/T127A/L128A}$ *-yEVenus::URA3, hta2::Hta2-mCherry::LEU2*	this paper
Wild-type +pJT29	yJT332_1	BY4741+pRS416 pGAL1 *rad50*$^{L116A/I119A/T127A/L128A}$-tCYC1	this paper

Strain name	Strain number	Genotype	Source
Wild-type +pJT28	yJT333_1	BY4741+pRS416 pGAL1-*rad50*$^{N121A/D124N}$-tCYC1	this paper
Wild-type +pJT27	yJT334_1	BY4741+pRS416 pGAL1-*rad50*-tCYC1	this paper
Wild-type +pNPe35	yJT335_1	BY4741 +pRS416 pGAL1	this paper
Rad50^{lo}	yJT336_1	BY4741 *rad50::kanMX, ura3::rad50*$^{L116A/I119A/T127A/L128A}$	this paper
Rad50^{wt}	yJT337_2	BY4741 *rad50::kanMX, ura3::rad50*	this paper
Rad50^{ho}	yJT338_1	BY4741 *rad50::kanMX, ura3::rad50*$^{N121A/D124N}$	this paper
rad50Δ +pNPe35	yJT346_1	BY4741, *rad50::kanMX* +pRS416 pGAL	this paper
Wild-type	yNA1594/yNA1595	As BY4741	single colonies derived from BY4741 wild-type
rad50Δ	yNA1600/yNA1601	As yJT305	single colonies derived from yJT305
Rad50^{wt}	yNA1909/yNA1910	As yJT337_2	single colonies derived from yJT337_2
Rad50^{ho}	yNA1911/yNA1912	As yJT338_1	single colonies derived from yJT338_1
Rad50^{lo}	yNA1913/yNA1914	As yJT336_1	single colonies derived from JT336_1

Supplementary References

1. Sonn-Segev, A. *et al.* Quantifying the heterogeneity of macromolecular machines by mass photometry. *Nat. Commun.* **11**, 1772 (2020).
2. Mumberg, D., Mueller, R. & Funk, M. Regulatable promoters of Saccharomyces cerevisiae: comparison of transcriptional activity and their use for heterologous expression. *Nucleic Acids Res.* **22**, 5767–5768 (1994).
3. Wosika, V. *et al.* New families of single integration vectors and gene tagging plasmids for genetic manipulations in budding yeast. *Mol. Genet. Genomics* **291**, 2231–2240 (2016).
