# Supplementary material for: Mre11-Rad50 oligomerization promotes DNA double-strand break repair
Source: Nat Commun. 2022 May 2;13:2374. doi: 10.1038/s41467-022-29841-0 (PMC9061753; doi:10.1038/s41467-022-29841-0)
Supplement: Supplementary file 3 — Description of additional Supplementary File [file 41467_2022_29841_MOESM3_ESM.pdf]

Descriptions of Additional Supplementary Files

Supplementary Software : Exemplary custom-written Python code for the analysis of mass photometer data.
