# Supplementary material for: Mre11-Rad50 oligomerization promotes DNA double-strand break repair
Source: Nat Commun. 2022 May 2;13:2374. doi: 10.1038/s41467-022-29841-0 (PMC9061753; doi:10.1038/s41467-022-29841-0)
Supplement: Supplementary file 5 — Reporting Summary [file 41467_2022_29841_MOESM5_ESM.pdf]

nature portfolio

Corresponding author(s): Prof. Dr. Matthias Peter
Dr. Petr Cejka

Last updated by author(s): Mar 18, 2022

Reporting Summary

Nature Portfolio wishes to improve the reproducibility of the work that we publish. This form provides structure for consistency and transparency in reporting. For further information on Nature Portfolio policies, see our Editorial Policies and the Editorial Policy Checklist.

Statistics

For all statistical analyses, confirm that the following items are present in the figure legend, table legend, main text, or Methods section.

n/a	Confirmed	
☐	☒	The exact sample size (*n*) for each experimental group/condition, given as a discrete number and unit of measurement
☐	☒	A statement on whether measurements were taken from distinct samples or whether the same sample was measured repeatedly
☐	☒	The statistical test(s) used AND whether they are one- or two-sided<br>*Only common tests should be described solely by name; describe more complex techniques in the Methods section.*
☒	☐	A description of all covariates tested
☒	☐	A description of any assumptions or corrections, such as tests of normality and adjustment for multiple comparisons
☐	☒	A full description of the statistical parameters including central tendency (e.g. means) or other basic estimates (e.g. regression coefficient) AND variation (e.g. standard deviation) or associated estimates of uncertainty (e.g. confidence intervals)
☐	☒	For null hypothesis testing, the test statistic (e.g. *F*, *t*, *r*) with confidence intervals, effect sizes, degrees of freedom and *P* value noted<br>*Give P values as exact values whenever suitable.*
☒	☐	For Bayesian analysis, information on the choice of priors and Markov chain Monte Carlo settings
☒	☐	For hierarchical and complex designs, identification of the appropriate level for tests and full reporting of outcomes
☒	☐	Estimates of effect sizes (e.g. Cohen's *d*, Pearson's *r*), indicating how they were calculated

Our web collection on statistics for biologists contains articles on many of the points above.

Software and code

Policy information about availability of computer code

Data collection	TEM micrographs were acquired with a FEI Morgagni 268 microscope using the Morgagni User Interface 3.0 and iTEM 5.2 softwares;<br>Gel images were collected using a Typhoon FLA 9000 imaging system (GE Healthcare) with Typhoon FLA 9500 software;<br>Mass photometry data was collected using a OneMP device (Refeyn Ltd) with AcquireMP (Refeyn Ltd) 2.3.0 software;<br>ThT measurements and growth assays were measured using a CLARIOstar plate reader (BMG Labtech) and 5.40 R3 software;<br>Blots and yeast plate images were taken using a Vilber Fusion FX6 system and FusionCapt Adv FX7 software;<br>Live-cell imaging was performed using a Visitron system controlled by VisiVIEW software 5.0.0;<br>Flow cytometry analysis was performed on the BD LSRFortessa flow cytometer (BD Biosciences) using the BD FACSDiva software (v9.0.1);<br>Telo-PCR images were taken with the ChemiDoc Touch Imaging System (BioRad);
Data analysis	TEM images were analyzed using Fiji/ImageJ 1.52t, Excel 2016 and GraphPad Prism 9;<br>Gels were quantitated with Fiji/ImageJ 1.52t and Excel 2016, and statistically analyzed with GraphPad Prism 9;<br>Mass photometry data was analyzed using DiscoverMP (Refeyn Ltd, version 2.3.0), OriginPro 2017 and a custom-written Python program (in Supplementary files);<br>Statistical analysis of ThT measurements and growth curves was performed with GraphPad Prism 9;<br>Live-cell imaging was analyzed using Fiji/ImageJ 1.52t, YeastQuant 2021, Matlab R2017b and GraphPad Prism 9;<br>Flow cytometry data was analyzed using FlowJo (v10.8.0) software;<br>The analysis of the mean telomere length and distribution was executed using the ImageLab software version 5.2.1 (BioRad) and the graphs were generated using Graph Pad Prism 9 software;<br>The crystal structure of Rad50 was visualized using Pymol 1.4.1;<br>Protein sequence alignment was assisted using the HHPred MPI Bioinformatics Toolkit website of Max Planck Institute for Developmental Biology, Tübingen (https://toolkit.tuebingen.mpg.de/tools/hhpred);<br>Graphs were plotted and statistically analyzed using GraphPad Prism 9;

Figure presentation: Adobe Illustrator 2022, Adobe Indesign 2022, Adobe Photoshop 2022, PowerPoint 2016.

For manuscripts utilizing custom algorithms or software that are central to the research but not yet described in published literature, software must be made available to editors and reviewers. We strongly encourage code deposition in a community repository (e.g. GitHub). See the Nature Portfolio guidelines for submitting code & software for further information.

Data

Policy information about availability of data

All manuscripts must include a data availability statement. This statement should provide the following information, where applicable:

- Accession codes, unique identifiers, or web links for publicly available datasets
- A description of any restrictions on data availability
- For clinical datasets or third party data, please ensure that the statement adheres to our policy

The Rad50 crystal structure was obtained from the RCSB Protein Data Bank (PBD: 5DAC, https://www.rcsb.org/);
Rad50 patient mutations were found in the ClinVar and MedGen Databases (https://www.ncbi.nlm.nih.gov/clinvar/) and (https://www.ncbi.nlm.nih.gov/medgen/).

Relevant data produced or analyzed in this paper are included in this article and its Supplementary information. Source data are provided with this paper including uncropped images of gels and blots. The data supporting this study are available from the corresponding authors upon reasonable request.
An exemplary custom-written Python code for the analysis of mass photometer data is provided with this study as Supplementary Data file 1.

Field-specific reporting

Please select the one below that is the best fit for your research. If you are not sure, read the appropriate sections before making your selection.

[x] Life sciences [ ] Behavioural & social sciences [ ] Ecological, evolutionary & environmental sciences

For a reference copy of the document with all sections, see nature.com/documents/nr-reporting-summary-flat.pdf

Life sciences study design

All studies must disclose on these points even when the disclosure is negative.

Sample size	Sample size and number of replicates were not pre-determined and chosen based on common practice in the field or what was practical to do (see also recent publications from Peter/Cejka/Seidel/Luke laboratories, e.g. Acharya et al., Nature Comm. 2021, PMID: 34764291). Thus at least 2-3 independent, biological replicates were performed per panel shown. Of plasmids and strains, multiple clones were tested. For images that were not quantified, but were more for visualisation, 2 independent experiments were performed. Note that the single molecule imaging data are also orthogonally validated using alternative, in most cases population-based measurements.
Data exclusions	No data were excluded except for experiments/gels or gel lanes with technical problems, e.g. contaminated plates, broken gels, loading problem in certain gel lanes etc. that invalidated the analysis.
Replication	For all panels, at least 2-3 independent experiments were measured and data could thereby be successfully replicated and reproduced, except when the above-mentioned technical failures occurred.
Randomization	Randomization or covariates were not applicable and relevant for the types of experiments performed, as no case study/control study was done with individual human or animal (e.g. mice) participants involved.
Blinding	For manual counting of cells with nuclear Rad50 foci using Cell Counter, one investigator was blinded for analysis to avoid potential analysis bias by the person acquiring the images. The blinded investigator and the experimentalist acquiring the images did, however, independently of each other reach the same conclusions. All other experiments could be either objectively quantified or were only presented for visualisation, thus were not prone to experimentalist bias. Moreover, the types of data shown in this study prevented blinding in most cases as presentation was important, e.g. loading order in gels.

Reporting for specific materials, systems and methods

We require information from authors about some types of materials, experimental systems and methods used in many studies. Here, indicate whether each material, system or method listed is relevant to your study. If you are not sure if a list item applies to your research, read the appropriate section before selecting a response.

Materials & experimental systems

n/a	Involved in the study	
	x	Antibodies
	x	Eukaryotic cell lines
x		Palaeontology and archaeology
x		Animals and other organisms
x		Human research participants
x		Clinical data
x		Dual use research of concern

Methods

n/a	Involved in the study	
x		ChIP-seq
	x	Flow cytometry
x		MRI-based neuroimaging

Antibodies

Antibodies used

Mouse monoclonal anti-Rad53 antibody (clone EL7.E1, Abcam, ab166859, 1:1'000); mouse monoclonal anti-Pgk1 antibody (clone 22C5D8, Invitrogen, 459250, 1:10'000); goat anti-mouse (GAM)-HRP conjugate secondary antibody (BioRad, 170-5047, 1:2'000); rabbit polyclonal anti-yeast Rad50 antibody (ThermoScientific, PA5-32176, 1:1'000); mouse monoclonal anti-Tubulin antibody (clone B-5-1-2, Sigma-Aldrich, T5168, 1:20'000); monoclonal mouse anti-MBP antibody (clone MBP-17, Abcam, ab49923, 1:1'000);
goat anti-rabbit HRP-conjugated secondary antibody (BioRad, 170-6515, 1:2'000); anti-mouse HRP-conjugated secondary antibody (BioRad, 170-6516, 1:2'000).

Validation

The Rad50 antibody was validated for yeast lysates by the manufacturer (https://www.thermofisher.com/antibody/product/RAD50-Antibody-Polyclonal/PA5-32176) and for purified, recombinant protein by us; all other antibodies are well-characterized, commercial and standard antibodies, see manufacturer validations here:
https://www.abcam.com/rad53-antibody-el7e1-ab166859.html
https://www.thermofisher.com/antibody/product/PGK1-Antibody-clone-22C5D8-Monoclonal/459250
https://www.sigmaaldrich.com/CH/de/product/sigma/t5168
https://www.abcam.com/maltose-binding-protein-antibody-mbp-17-hrp-ab49923.html

Eukaryotic cell lines

Policy information about cell lines

Cell line source(s)

S. cerevisiae strain BY4741 wild-type from OpenBiosystems was used to create all other strains listed in the Suppl. Table and used in this study.

Authentication

For BY4741 wild-type provided by OpenBiosystems

Mycoplasma contamination

Yeast strains were not tested for mycoplasma contamination.

Commonly misidentified lines
(See ICLAC register)

The BY4741 strains are not among the misidentified lines.

Flow Cytometry

Plots

Confirm that:

- [x] The axis labels state the marker and fluorochrome used (e.g. CD4-FITC).
- [x] The axis scales are clearly visible. Include numbers along axes only for bottom left plot of group (a 'group' is an analysis of identical markers).
- [x] All plots are contour plots with outliers or pseudocolor plots.
- [x] A numerical value for number of cells or percentage (with statistics) is provided.

Methodology

Sample preparation

Untagged Rad50 strains were grown to exponential phase at 30 °C and treated with 0.2 M Hydroxyurea, HU (Sigma Aldrich, H8627). Cells of exponential samples, 30 min- and 60 min-treated samples with OD600 = 0.5 were fixed in 70 % ethanol overnight. Pellets were treated with RNase A (ThermoFisher Scientific, 10753721) at 37 °C for 2 h and Proteinase K (Biofroxx, 1151ML010) at 50 °C for 2 h in 50 mM Tris-HCl pH 7.5 buffer. The cell suspension was sonified using a Branson sonifier 450 for 5 sec with output control 1 and duty cycle constant. Then, cells were stained with 2.4 µM SYTOX Green (ThermoFisher Scientific, 1076273).

Instrument

Measurement was performed on the BD LSRFortessa flow cytometer (BD Biosciences)

Software

BD FACSDiva software (v9.0.1)

Cell population abundance	18'000-20’000 events were recorded.
Gating strategy	Analysis was performed with FlowJo (v10.8.0) using the following gating strategy: From the main population in FSC-A vs. SSC-A, doublets were excluded in the Sytox-Green A vs. W channel, and DNA content was assessed in the histogram of the Sytox-Green-A channel (Ex 488nm, 530/30BP).

☒ Tick this box to confirm that a figure exemplifying the gating strategy is provided in the Supplementary Information.
